# Clinical characteristics and risk factors of lower extremity amputation in patients with diabetic foot

**DOI:** 10.12669/pjms.38.8.5635

**Published:** 2022

**Authors:** Linru Wang, Qiang Li, Xiao Chen, Zhaowei Wang

**Affiliations:** 1 Linru Wang, Vascular Surgery Department, Qingdao Hiser Medical Group, Qingdao, Shandong Province, 266033, China; 2 Qiang Li, Vascular Surgery Department, Qingdao Hiser Medical Group, Qingdao, Shandong Province, 266033, China; 3 Xiao Chen, Vascular Surgery Department, Qingdao Hiser Medical Group, Qingdao, Shandong Province, 266033, China; 4 Zhaowei Wang, Vascular Surgery Department, Qingdao Hiser Medical Group, Qingdao, Shandong Province, 266033, China

**Keywords:** Risk factors, Lower extremity amputation, Diabetic foot

## Abstract

**Objectives:**

To investigate the risk factors of lower limb amputation, and help physicians better understand the clinical characteristics of patients with diabetic foot, and make treatment strategies for these patients correctly.

**Methods:**

In this study, the inpatients with diabetic foot treated in our hospital form January 2013 to February 2021 were reviewed retrospectively. The patients were divided into amputation and conservative treatment groups. The variables of the patients, consisting of age, gender, smoking history, alcohol use, diabetes and ulcer duration, ulcer size, Wagner classification, ankle brachial index, previous amputation history, laboratory data, and medical comorbidities including hypertension, coronary artery disease, peripheral arterial disease, chronic renal insufficiency, retinopathy, and sequelae of cerebral infarction were selected for analysis to determine the risk factors of lower limb amputation.

**Results:**

In this study, a total of 856 patients with diabetic foot were enrolled, in which 487 patients received amputation surgeries, and the amputation rate was 56.9%. There were significant differences between the two groups in gender (p=0.014), smoking history (p=0.011), ulcer duration (p=0.023), ulcer size (p=0.000), Wagner classification (p=0.000), ABI (p=0.031), peripheral arterial disease (p=0.000), HDL-C (p=0.013), osteomyelitis (p=0.000), and fibrinogen (p=0.001). A stepwise multiple logistic regression analysis revealed that male gender (p=0.003), larger ulcer size (p=0.001), higher Wagner classification grades (p=0.002), higher rate of peripheral arterial disease (p=0.02) and osteomyelitis (p=0.0001), and increased fibrinogen level (p=0.004) were independent risk factors of lower limb amputation in patients with diabetic foot.

**Conclusion:**

The diabetic foot patients with male sex, larger ulcer size, higher grade of Wagner classification, peripheral arterial disease or higher fibrinogen level may face higher risk of lower limb amputation.

## INTRODUCTION

As a complex chronic metabolic disorder, the prevalence of Type-2 Diabetes Mellitus (T2DM) is sharply increasing^1^, and it is estimated that in the year of 2030, the prevalence of T2DM may be increased to 4.4% in the world.^2^ T2DM may lead to many serious complications, among these complications diabetic foot ulcers are one of the most common problems, which can cause prolonged hospitalization, long-term and broad-spectrum antimicrobial therapy, amputation of lower limb, and severe infections.^3^ In many cases, amputation may have to be performed as a final treatment^4^, so amputation of lower limb is a major grave outcome of diabetic foot, which influences the quality of life of the patients adversely. Subsequently, analyzing the risk factors of lower extremity amputation and trying to prevent its occurrence become an extensive concern in medical fields.

Some surgeons have investigated the risk factors of lower extremity amputation in patients with diabetic foot. In a study of 225 patients admitted with diabetic foot-related complications, there were 51 patients who were advised foot amputation, the rate of amputation was 22.5%, and Nanwani found the crucial risk factors include male gender, smoking, hyperlipidemia, hypertension, cardiac history, and the coexistence of diabetic nephropathy and retinopathy.^4^ In another study of 351patients with diabetic foot ulcers, 170 received amputation surgeries and the rate of amputation was high up to 48.4%, and Jun Ho Lee concluded that osteomyelitis, peripheral artery disease, chronic kidney disease, ulcer size, and forefoot ulcer location were risk factors of amputation in diabetic foot patients.^5^ In addition, some other authors have also reported the incidence and risk factors of amputation in patients with diabetic foot ulcer.^2^,^6^ In general, these studies advocated that multifactorial causes and socioeconomic status can lead to the development of a diabetic foot ulcer and subsequent amputation surgeries.^5^ However, up to now, there was a high variability in terms of the reported risk factors between studies in the published literature. Moreover, most of the published studies had a small sample size, which may cause various results in this issue. We believe that a large scaled clinical study may be helpful in clarifying the risk factors of lower limb amputation in patients with diabetic foot ulcer.

Therefore, in this study we reviewed retrospectively the patients with diabetic foot treated in our hospital from January 2013 to February 2021, and the objectives of our study were to investigate the risk factors of lower limb amputation, to help physicians better understand the clinical characteristics of T2DM patients with diabetic foot, and make treatment strategies for these patients correctly.

## METHODS

In this study, the inpatients with diabetic foot treated in our hospital form January 2013 to February 2021 were reviewed retrospectively. This study was approved by the ethical committee of our hospital on March 10, 2021.

### Inclusion criteria:

- Patients who were diagnosed with diabetic foot,
- The patients whose age were greater than 18 years.

In the current study, the diagnosis of diabetic foot was determined according to the diagnostic criteria of diabetic foot in the 2002 edition of Endocrinology^7^, and the foot ulcers were graded based on Wagner criteria (Table-I)^8^, and amputation was defined as surgery going beyond the toe level.^5^ Patients with ulcer and gangrene resulted from other reasons, or traumatic amputations were excluded from this study.

**Table-I:** Wagner criteria for diabetic foot ulcers.

Grade	Description
0	Skin lesions absent, hyperkeratosis below or above bony prominences
1	Skin and immediate subcutaneous tissue are ulcerated
2	Lesions are deeper and may penetrate to tendon, bone or joint capsule
3	Deep tissues are always involved, osteomyelitis may be present
4	Gangrene of some portion of the toes or forefoot
5	The entire foot is gangrenous

The patients’ medical records were examined to collect related information. According to previously published studies, the variables, consisting of age, gender, smoking history, alcohol use, diabetes and ulcer duration, ulcer size, Wagner classification, ankle brachial index (ABI), previous amputation history, laboratory data including white blood cell count, plasma albumin, high-sensitivity C-reactive protein (hsCRP), hemoglobin (Hb), glycosylated hemoglobin A1c (HbA1c), high density lipoprotein cholesterol (HDL-C), low density lipoprotein cholesterol (LDL-C), fibrinogen (Fib), activated partial thromboplastin time (APTT)), and medical comorbidities including hypertension, coronary artery disease (CAD), peripheral arterial disease (PAD), chronic renal insufficiency (CRI), retinopathy, and sequelae of cerebral infarction were selected for analysis to determine the risk factors of lower limb amputation. In the above mentioned variables, the ulcer size was defined as the longest diameter in centimeters.^5^ Ankle-brachial index (ABI) was determined through dividing the systolic blood pressure of the ankle by the systolic blood pressure of the upper arm of the affected side,^5^ and hypertension, coronary artery disease, peripheral arterial disease, chronic renal insufficiency (CRI), and sequelae of cerebral infarction were defined based on previously documented diagnoses in medical records.

In this study, categorical data were represented by percentages, and continuous variables were expressed as means ± standard deviation. Student t-test or nonparametric rank sum test was carried out to compare the continuous variables, and chi-square test was conducted to analyze the categorial data, and the risk factors for lower limb amputation were determined through stepwise multiple logistic regression analysis. The statistical analysis was performed based on IBM SPSS version 22.0 (IBM Corp, Armonk, NY, USA), and p values less than 0.05 were considered significant.

## RESULTS

In this study, a total of 856 patients with diabetic foot were enrolled, in which 487 patients received amputation surgeries, 369 received conservative treatments, and the amputation rate was 56.9%. The patients were divided into amputation and conservative treatment groups based on the treatment methods, i.e. 487 patients were assigned to amputation group and 369 to conservative treatment group. The basic characteristics of the two groups are summarized in Table-I.

In terms of age (p=0.753), previous amputation history (p=0.884), alcohol use (p=0.696), diabetes duration (p=0.932), white blood cell count (p=0.683), plasma albumin (p=0.752), hsCRP (p=0.492), Hb (p=0.662), HbA1c (p=0.179), LDL-C (p=0.892), and medical comorbidities including hypertension (p=0.338), CAD (p=0.183), CRI (p=0.645), retinopathy (p=0.564) and sequelae of cerebral infarction (p=0.495), there were no significant differences between the two groups (Table-II).

**Table-II:** Baseline characteristics of the two groups.

Variables	Amputation (n=487)	Conservative treatment (n=369)	p value
Age (year)	63.8±11.5	61.6±12.3	0.753
Gender (male/female)	302/185	198/171	0.014
Previous amputation history(Y/N)	85/402	63/306	0.884
Smoking history (present or past)	251/236	158/211	0.011
Alcohol use (Y/N)	379/108	283/86	0.696
Diabetes duration year	18.4±9.5	17.9±10.1	0.932
Ulcer duration (month	6.8±3.1	3.7±2.5	0.023
Ulcer size (>2cm, n)	309	153	0.000
Wagner classification(≥Grade 3, n)	388	79	0.000
Ankle brachial index	1.0±0.2	1.2±0.3	0.031
Albumin (g/dl)	3.8±1.1	3.9±1.3	0.752
WBC(x10^3^/μl)	10.9±1.6	9.7±1.7	0.683
High-sensitivity C-reactive protein	6.8±8.4	6.3±7.9	0.492
Hemoglobin (Hb, g/dl)	12.21.8	11.9±1.6	0.662
Glycosylated hemoglobin A1c (HbA1c, %)	8.4±3.7	8.3±3.4	0.179
High density lipoprotein cholesterol (mg/dl)	39.5±13.9	35.3±12.8	0.013
Low density lipoprotein cholesterol (mg/dl)	87.5±36.4	91.6±38.1	0.892
Fibrinogen (Fib, mg/dl)	476±129	381±97	0.001
Hypertension (Y/N)	383/104	280/89	0.338
Osteomyelitis (Y/N)	362/125	89/280	0.000
Coronary artery disease (Y/N)	256/231	177/192	0.183
Peripheral arterial disease (Y/N)	289/198	168/201	0.000
Chronic renal insufficiency (Y/N)	98/389	79/290	0.645
Sequelae of cerebral infarction (Y/N)	76/411	64/305	0.495
Retinopathy (Y/N)	138/349	98/271	0.564

However, as to gender(p=0.014), smoking history(p=0.011), ulcer duration(p=0.023), ulcer size (p=0.000), Wagner classification (p=0.000), ABI (p=0.031), peripheral arterial disease (p=0.000), HDL-C (p=0.013), osteomyelitis (p=0.000), and fibrinogen (p=0.001), there were significant differences between the two groups (p<0.05), and the amputation group had significantly lower HDL-C level, larger ulcer sizes, longer ulcer durations, higher Wagner classification grades, increased fibrinogen levels, higher prevalence of PAD, osteomyelitis and previous amputation (Table-II).

**Fig.1:**
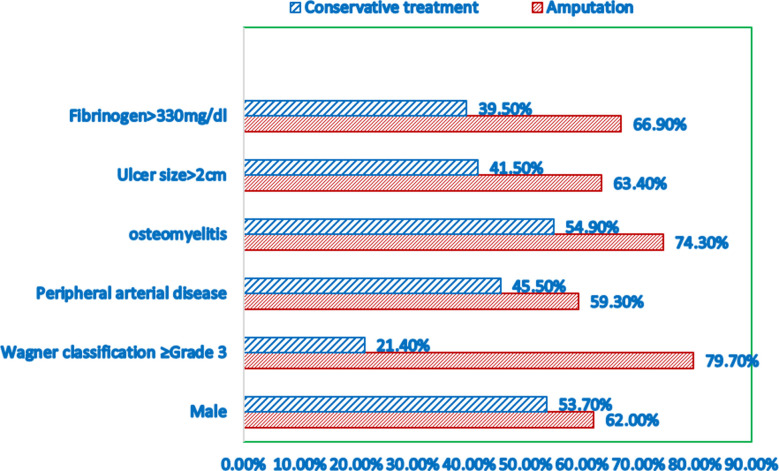
The distribution of gender, ulcer size, Wagner classification grades, rate of peripheral arterial disease, osteomyelitis, and increased fibrinogen level in the two groups.

In addition, a stepwise multiple logistic regression analysis was performed to detect the independent risk factors, and the results revealed that male gender (p=0.003), larger ulcer size (p=0.001), higher Wagner classification grades (p=0.002), higher rate of peripheral arterial disease (p=0.02) and osteomyelitis (p=0.0001), and increased fibrinogen level (p=0.004) were independent risk factors of lower limb amputation in patients with diabetic foot (Table-III, Fig.1).

**Table-III:** Multivariate logistic regression analysis of diabetic foot amputation.

Variables	p value	Odd ratios	95% CI
Male gender	0.003	3.582	1.765-7.684
Ulcer size >2cm	0.001	4.119	2.304-9.192
Wagner classification	0.002	3.724	1.098-6.247
Peripheral arterial disease	0.02	6.752	4.081-8.453
Osteomyelitis	0.0001	4.876	3.695-5.803
Fibrinogen	0.004	1.342	1.103-5.449

## DISCUSSION

In the current study, a total of 856 patients were enrolled to investigate the clinical characteristics and risk factors of amputation in patients with diabetic foot. In this study, the amputation rate was 56.9%. In the previously published literature, the authors reported different rates of lower limb amputation. Nanwani reported the rate of amputation was 22.5%,^4^ Jun Ho Lee reported the rate of amputation was 48.4%,^5^ and Uysal reported the rate was 33.2%.^3^ Obviously, in this study, the amputation rate was relatively higher than above-mentioned studies. Many factors, such as patient sources, inclusion criterion and admission standard, may affect the rate of amputation, and the higher amputation rate in the current study may be attributed to admission standards, i.e., only severe cases were hospitalized.

We found in the amputation group there were more male patients, and male gender was found to be an independent risk factor of amputation in patients with diabetic foot. In previously published studies, Lin and Nanwani also concluded the same conclusion.^4^,^9^ However, in Uysal’ s study, gender was not an independent predictor of amputation.^3^ Moreover, it is important to note that in a study of 131 patients, Kogani found female gender was an independent risk factor of amputation,^10^ the viewpoint of which was opposite to the current study and Lin’s study. In our opinion, gender may be influenced by many other factors, such as culture, nation, and dietary habit, which may affect the outcomes of clinical studies. Despite of this, as a meta-analysis study, Lin’s conclusion was drawn based on 21 studies and 6505 participants, which may be worth accepting.

Both Li and our study focused on the influence of fibrinogen level on the treatment selection in patients with diabetic foot. In a retrospective study of 152 patients with diabetic foot, Li found the mean fibrinogen level in diabetic foot patients with Wagner classification grade≥3 was significantly higher than those with grades 1 and 2, and revealed that fibrinogen level was a biomarker for assessing the disease severity and monitoring the disease progression in patients with diabetic foot, and the predictive superiority of fibrinogen was attributed to its more stable nature in comparison with CRP.^11^ In the current study, we obtained the similar results as Li’s study, and found the fibrinogen level in the amputation group was significantly higher than that in the conservative group, and fibrinogen was found to be independent risk factor of amputation. Thus, our study demonstrated that fibrinogen level can reflect the severity of diabetic foot. In this study we didn’t evaluate the association between fibrinogen level and Wagner classification, but the Wagner classification was also found to be an independent factor of amputation. In addition, hs-CRP was used as variable in this study, but between the two groups there was no significant difference in this indicator. Subsequently, our study further proved the findings in Li’s study.

In addition, we found the amputation risk was seven, five, and three times higher in patients with PAD, osteomyelitis, and higher ulcer size, compared with those without. In the multiple logistic regression analysis, these three factors were found to be independent predictors of amputation in patients with diabetic foot, which were consistent with the outcomes of many clinical studies.^5^,^9^,^12^ However, some factors, such as WBC count, ulcer duration, and coexistence of diabetic retinopathy, were found to be independent factors in other studies,^4^,^5^,^9^ but didn’t have significant differences between amputation and conservative treatment groups in the current study. Moreover, in Karakas’ study, IL-6 was regarded to have predictive value for lower extremity amputation in patients with diabetic foot, but we didn’t analyze this factor in the current study^13^. We think, these facts demonstrated that many factors may affect the outcomes of these clinical studies, and some complex correlations may be available among different factors.

In short, this study revealed that male gender, larger ulcer size, higher Wagner classification grades, higher rate of peripheral arterial disease and osteomyelitis, and increased fibrinogen level were independent risk factors of lower limb amputation in patients with diabetic foot.

### Limitations of the study:

First, it is a retrospective design, many potential risk factors were not analyzed due to restricted data availability. Second, the enrolled patients came from one single hospital, the rate of amputation in patients with diabetic foot usually show variety from hospital to hospital^3^, and patient source of a single hospital may also affect the final results. Thus, more studies need to be carried out in the future.

## CONCLUSION

The diabetic foot patients with male sex, larger ulcer size, higher grade of Wagner classification, peripheral arterial disease or higher fibrinogen level may face higher risk of lower limb amputation.

### Authors’ Contribution

**QL & LRW:** conceived, designed the project and performed the statistical analysis,

**QL, LRW, XC & ZWW:** did data collection and wrote the manuscript,

**QL & LRW:** did review and final approval of the manuscript.
